# Prevalence of dental carries and its association with breastfeeding duration among young children in Addis Ababa, Ethiopia

**DOI:** 10.1186/s12889-024-19044-1

**Published:** 2024-06-06

**Authors:** Marta Yemane Tesfay, Tefera Darge Delbiso

**Affiliations:** Department of Public Health Nutrition and Dietetics, School of Public Health, Addis Ababa University, Addis Ababa, Ethiopia

**Keywords:** Dental caries, Breastfeeding duration, Oral health, Deft index

## Abstract

**Background:**

Breastfeeding is a crucial feeding practices that significantly contributes to the healthy development of children. However, the effect of breastfeeding duration on caries risk is unclear, as different studies have found different results. This study aims to assess the prevalence of dental caries and its association with breastfeeding duration among young children aged 12–36 months in selected health facilities of Addis Ababa, Ethiopia.

**Methods:**

A cross-sectional study among 380 children aged 12–36 months from 11 health centers in Addis Ababa was conducted. Questionnaires and dental examinations were used to collect data. Dental caries was measured by the deft (decayed–extracted–filled teeth) index. Binary logistic regression was used to assess the association between dental caries and duration of breastfeeding, after adjusting for the confounders.

**Results:**

The prevalence of dental caries was 53.4% (95% CI: 48.3, 58.5%), with 13.7% having high caries and 39.7% having low caries. Breastfeeding duration was not significantly associated with dental carries, after adjusting for confounders. However, sugar intake, older age, mothers’ unemployment, and not being in marital union are risk factors for dental caries development.

**Conclusions:**

Promotion of healthy diet, especially limiting intake of sugar and sweets, and integration of oral health into primary health care programs are recommended. Further research using longitudinal design or meta-analysis is recommended to establish more concise evidence on the association between breastfeeding duration and dental caries.

## Background

Dental caries, a tooth disease that affects the primary teeth of infants and toddlers, is one of the most common noncommunicable diseases worldwide. It is preventable, but it remains a lifelong progressive and cumulative chronic condition [1, 2]. Globally, 2.5 billion people are affected by untreated dental caries in permanent teeth and 573 million children had untreated caries in deciduous teeth [3]. The prevalence of dental caries varies across regions, population groups, socioeconomic status, and biological, behavioral and psychological factors [4–8]. A recent meta-analysis shows that the prevalence of early childhood caries was 48% globally and 30% in the African content [9]. Dental caries is costly to health care and negatively affects young children’s quality of life, well-being, growth and development [1, 2, 10, 11] and is linked with feeding practices, oral hygiene, socio-demographic factors and chronic illness [12].

Breastfeeding is one of the feeding practices which contributes to healthy development of children. The World Health Organization (WHO) and UNICEF recommend to breastfeed exclusively for 6 months and continue with complementary feeding until two years and beyond [13]. Breast milk for infants and mothers provides health, economic, and psychological benefits. On the other hands, breast milk has more cariogenic properties that is responsible for dental caries because it contains more carbohydrates and less calcium, phosphorus, and protein compared to cow’s milk [14].

Breastfeeding and dental caries in young children have a complex association that has been studied but with contradictory results. Some researchers argue that prolonged breastfeeding contributes to caries development [15–19] others claimed ever breastfeeding is protective against caries [20], and some found no association [21]. Children breastfed for 6–24 months had an increased risk of dental caries compared to those breastfed for less than 6 months [15, 16]. In another study, breastfeeding for more than 12 months significantly increased caries risk compared with those breastfed for less than 12 months [20]. A similar study conducted in southern Brazil found that children who were breastfeed for ≥ 24 months had a 2.4 times higher risk of having early childhood caries compared to those breastfed up to 12 months of age [17]. The 2016 Lancet review described dental caries as the only negative health outcome related to prolonged breastfeeding [22].

While it is widely acknowledged that dental caries has negative impact on young children’s quality of life, research on the risk factors specific to Ethiopia remains limited; we found only two published studies [23, 24]. Notably, the association between breastfeeding duration and dental caries has not been thoroughly investigated among children 12–36 months old in Ethiopia. This raises the question of what constitutes the best form of infant feeding to prevent dental caries and promote optimal dental health. Overall, little attention is given to dental health in Ethiopia; for example, the country does not have national oral health policy and strategy [25]. This study aimed to provide evidence by estimating the prevalence of dental caries and assessing its association with breastfeeding duration among children aged 12–36 months in Addis Ababa, Ethiopia.

## Methods

### Study design, setting, and population

A facility-based cross-sectional study design was conducted from September–December 2021 in selected primary health centers at Addis Ababa, the capital city of Ethiopia. The Addis Ababa city has 13 public hospitals and 112 health centers during the study period. The study population comprised of young children age between 12 and 36 months attending the under five units (immunization and growth monitoring units) at the selected primary health centers. Children who had at least two teeth erupted were included in the study. Children with major illnesses that may affect the assessment of caries condition were excluded from the study.

### Sample size and sampling procedure

To estimate the prevalence of dental carries among young children, we used a single population proportion sample size estimation formula [26] considering the following assumptions: the prevalence of dental caries from an earlier study in Brazil was 33.8% [27], a margin of error 5%, and confidence level of 95%. The estimated sample size becomes 345. We added 10% contingency for potential non-response and the final sample size becomes 380 children. For assessing the association between breastfeeding duration and dental carries, we used a double population proportion sample size estimation formula [26] considering 80% power and 95% confidence level. Based on findings from a previous study in India [19], among children who breasted for less than 12 months, 53.6% had no dental carries while 15.4% had carries. Inputting these values in the formula and adding 10% contingency for non-response, the total sample was calculated to be 126 children. However, the sample size estimated using single population formula is higher and thus we used 380 children aged 12–36 months as our final sample size.

We first identified the list of 112 health centers in Addis Ababa. We then randomly selected 11 health centers (10% of the total health centers in the city). Using the previous month patient flow data, we proportionally allocated the calculated sample size to each of the selected health centers. Then, we systematically selected every third child to include in the study until the allocated sample was reached in each of the selected health centers.

### Variables and measurements

The outcome variable – dental caries – was calculated using the WHO deft (decayed–extracted–filled teeth) and describes the prevalence of dental caries in children less than 36 months [28]. Each child’s deft score is derived by adding the number of primary teeth that have decayed, been extracted due to caries, and been filled. The deft score is then categorized into three: high caries (deft score of ≥ 4), low caries (deft score of 1–4), and caries free (deft score of zero). For the Binary logistic regression, it is re-categorized into two: have caries (deft score ≥ 1) and caries free (deft score of zero).

The exposure variable – breastfeeding duration – is the length of time for any breastfeeding, including nursing through exclusive breastfeeding and complementary feeding until the cessation of feeding breast. Those who had never breastfed were classified as having 0 months of breastfeeding duration, whereas those breastfeeding during data collection were classified as having total breastfeeding duration equal to the child’s age. Finally, the breastfeeding duration was classified according to the WHO and UNICEF recommendations [13]: <6 months, 6–11 months, 12–24 months, and > 24 months.

The covariates were identified through literature review [5–8, 15–17, 19, 23, 29]. These includes feeding practices (feeding type, breastfeeding patterns, sleeping with breastfeeding, nocturnal breastfeeding, bottle-feeding and its contents, bottle-feeding at night, and pacifier use), intake of cariogenic snacks/drinks and table sugar, oral hygiene (child teeth cleaning status, frequencies, methods, onset, and cleaning support), mothers teeth cleaning frequencies and past oral case treatment, child characteristics (child’s sex and age), and parents characteristic (educational status, occupation, marital status, and household income).

### Data collection and quality assurance

The data collection tools were prepared by reviewing available literature and standard questionnaires that were already validated, including [7, 21, 30], the WHO infant and young children feeding practices manual [31], and the CDC oral health survey questionnaires [32]. Finally, structured questionnaires containing information on socio-demographic, feeding practices, oral health practices and cariogenic intake experiences was developed. The questionnaire was developed in English and translated into Amharic, commonly spoken languages in Addis Ababa. The questionnaire was pretested on 5% of the sample (20 children) in a health center other than the selected ones. It is then administered through face-to-face interview by qualified data collectors. Dental examination was conducted according to the WHO oral health survey procedure [28]. The data collection and dental examination was closely monitored by the principal investigator.

### Clinical examination

The dental examination was undertaken by fifth-year dental medicine students of Addis Ababa University and health professionals. They were further trained by experienced dentist to carry out the clinical examination, deft index, of the children according to the WHO oral health survey procedure [28]. Standardizations and calibration were done before data collection; the Cohen’s Kappa score of interexaminer reliability was calculated for deft scores and found to be 0.82, which shows better reliability. The children were examined by using disposable explorer, portable chair, dental mouth mirrors and flashlights. During the examination, the older children were seated on chair, and very young children were examined with the assistance of their caregivers using the “kneetoknee” technique. Gauze pads were used to clean and dry teeth surfaces before examination.

Dental caries was assessed by means of visual examination. Children having one or more decayed (cavitated or non-cavitated), extracted due to caries or filled teeth in any primary tooth were considered to have dental caries. Children with untreated caries were referred to hospitals for treatment, especially to Yekatit hospital.

### Data management and analysis

Data was collected through digital data collection tool, ODK application. The collected data was transferred into Excel, its completeness and consistency assessed, and then transported into SPSS for further analysis. We described the outcome, exposure, and confounding variables using frequencies and percentages and displayed them in tables and graphs. χ^2^ tests were used to make bivariate comparisons between the outcome (dental carries), and the exposure and confounding variables.

Dental caries prevalence was computed according to the deft index. Binary logistic regression analysis was conducted to assess the association between dental caries (caries free and have caries) and the exposure (breastfeeding duration), controlling for confounding variables. Clinically relevant variables and those significantly associated with the outcome variable at a 10% level of significant in the χ^2^ test of association were included in the regression model. Multicollinearity was tested using variance inflation factor (VIF) and found to be non-substantial (VIF < 3). The Hosmer and Lemeshow test indicated the model is a good-fit (*p* = 0.148). The statistical significance of the variables were decided based on *p*-value < 0.05. Crude and adjusted odds ratios (OR) and their respective 95% confidence intervals (CI) were reported for the final model.

### Ethical consideration

Ethical clearance was obtained from the School of Public Health, Collage of Health Science, Addis Ababa University Institutional Review Board. Each respondent (parent/gurdian) gave verbal informed consent during data collection after being given a brief explanation of the study objectives. Parental/guardian assent was obtained for the children. To preserve anonymity, names and other identifiers were not included in the questionnaire.

## Results

### Characteristics of study participants

A total of 380 mother-child pairs presented to the health center were identified and enrolled in the study. The mean (**±** standard deviation (SD)) age of the children was 25.1 (**±** 7.6 months) and 56.3% were 24–36 months old. About 16% of the mothers and 5% of the fathers had no formal education; 28% of the mothers and 13% of the fathers were unemployed; and 71% were married. Three-fourth of the households reported a monthly income of less than 10,000 ETB (~ 182 USD). Child age, mothers’ education, employment, marital status, and household income were significantly associated with dental carries at 10% level of significant and thus included in the Binary logistic regression (Table 1).

**Table 1:** Background characteristics of the study participants (*n* = 380) and its bivariate association with dental caries in Addis Ababa, Ethiopia, 2023

Characteristics	*n* (%)	Dental carries status, *n* (%)		*p*-value
		Caries free	Have caries	
Age of children, Mean (SD)	25.1 (± 7.6)			< 0.001
12–23 months	166 (43.7)	97 (54.8)	69 (34.0)	
24–36 months	214 (56.3)	80 (45.2)	134 (66.0)	
Sex of children				0.885
Female	184 (48.4)	85 (48.0)	99 (48.8)	
Mothers’ education				0.021
No formal education	60 (15.8)	33 (18.6)	27 (13.3)	
Primary or secondary	190 (50.0)	75 (42.2)	115 (56.7)	
Technical level and above	130 (34.2)	69 (39.0)	61 (30.0)	
Mothers’ employment				< 0.001
Employed (formal or informal)	274 (72.1)	143 (80.8)	131 (64.5)	
Current marital status				< 0.001
Married	270 (71.1)	145 (81.9)	125 (61.6)	
Not married	110 (28.9)	32 (18.1)	78 (38.4)	
Partners education				0.325
No formal education	18 (4.7)	9 (5.1)	9 (4.4)	
Primary or secondary	133 (35.0)	55 (31.1)	78 (38.4)	
Technical level and above	229 (60.3)	113 (63.8)	116 (57.1)	
Partners employment				0.406
Employed (formal or informal)	329 (86.6)	156 (88.1)	173 (85.2)	
Household monthly income (in Birr)				0.010
2,000–4,999	115 (30.3)	49 (27.7)	66 (32.5)	
5,000–9,999	182 (26.8)	72 (40.7)	100 (49.3)	
10,000 and above	93 (24.5)	56 (31.6)	37 (18.2)	

*p*-values are based on the χ^2^ test of association

### Feeding practices and duration

About 80% of the children were breastfed from the moment of birth; two-third were exclusively breastfed and nearly a fifth were breastfed for > 24 months as recommended. Slightly less than half of the children sleep with breastfeeding, 71% breastfeed ≥ 3 times at night, and 57% breastfed on demand. Roughly 37% of the children started solid food at the recommended 6-month of age. Of the bottle-feed children, 66% feed with cow’s milk with or without sugar and 45% slept with the bottle. 43% of the children use pacifiers. In the bivariate analysis, feeding type and breastfeeding duration are significantly associated with dental caries (Table 2).

**Table 2:** Feeding practices and duration among children 12–36 months old and its bivariate association with dental caries in Addis Ababa, Ethiopia, 2023

Characteristics	*N*	*n* (%)	Dental carries status, *n* (%)		*p*-value
			Caries free	Have caries	
Feeding type	380				0.027
Breastfeeding		302 (79.5)	132 (74.6)	170 (83.7)	
Bottle feeding		78 (20.5)	45 (25.4)	33 (16.3)	
Breastfeeding duration	380				0.090
< 6 months		146 (30.3)	66 (37.3)	80 (39.4)	
6–11 months		43 (11.3)	25 (14.1)	18 (8.9)	
12–24 months		119 (31.3)	47 (26.6)	72 (35.5)	
> 24 months		72 (18.9)	39 (22.0)	33 (16.3)	
Exclusive breastfeeding	349				0.534
< 6 months		115 (33.0)	50 (31.3)	65 (34.4)	
For 6 months		234 (67.0)	110 (68.8)	124 (65.6)	
Sleeping with breastfeeding at night	349	164 (47.0)	78 (44.1)	86 (42.4)	0.524
Nocturnal breastfeeding	349				0.312
< 3 times a night		81 (23.2)	36 (22.5)	45 (23.8)	
≥ 3 times a night		247 (70.8)	111 (69.4)	136 (72.0)	
Do not wake up at night		21 (6.0)	13 (8.1)	8 (4.2)	
Breastfeeding patterns	349				0.220
On demand		200 (57.3)	104 (65.0)	96 (50.8)	
Schedule		32 (9.2)	10 (6.3)	22 (11.6)	
Both		117 (11.7)	46 (28.8)	71 (37.6)	
Age solid food started	380				0.418
< 6 months		88 (23.2)	44 (24.9)	44 (21.7)	
At 6 months		142 (37.4)	60 (33.9)	82 (40.4)	
> 6 months		150 (39.5)	73 (41.2)	77 (37.9)	
Content of bottle-feeding	78				0.131
Formula milk		16 (20.5)	13 (28.9)	3 (9.1)	
Milk with sugar		27 (34.5)	15 (33.3)	12 (36.4)	
Milk without sugar		25 (32.1)	11 (24.4)	14 (42.4)	
Other than milk		10 (12.8)	6 (13.3)	4 (12.1)	
Sleeping with bottle	78	35 (44.9%)	78 (44.1)	86 (42.4)	0.524
Pacifier users	380	164 (43.2)	76 (42.9)	88 (43.3)	0.936

*p*-values are based on the χ^2^ test of association

### Cariogenic snacks/drinks consumption and oral hygiene practices of children and mother’s

Nearly 84% of the children consume sweet snacks at least once a day; fruit juice, biscuit and fast foods were commonly consumed snacks. Over half of the children consume table sugar. More than a quarter of the children practice teeth cleaning mainly starting from age 24–36 months (77%), and the majority clean their teeth once a day (86%) using fluoride toothpaste (55%). 70% of the mothers have been treated for oral problems in the past, and a tenth of them never clean their teeth. Sweet snacking, table sugar intake, and mothers’ own teeth cleaning are significantly associated with dental caries in the bivariate analysis and thus included in the multivariable regression (Table 3).

**Table 3:** Cariogenic snacks/drinks intake and oral hygiene practices among children 12–36 months old and its bivariate association with dental caries in Addis Ababa, Ethiopia, 2023

Characteristics	*N*	*n* (%)	Dental carries status, *n* (%)		*p*-value
			Caries free	Have caries	
**Cariogenic snacks/drinks intake**					
Sweet snacking	380				0.002
1–2 times a day		154 (40.5)	77 (43.5)	77 (37.9)	
≥ 3 times a day		165 (43.4)	62 (35.0)	103 (50.7)	
Do not snack		61 (16.1)	38 (21.5)	23 (11.3)	
Types of snacks	319				0.101
Fruit juice		81 (25.4)	32 (22.9)	49 (26.9)	
Biscuit		77 (24.1)	20 (14.3)	57 (31.3)	
Fast foods		74 (23.2)	52 (37.1)	22 (12.1)	
Candy		42 (13.2)	19 (13.6)	23 (12.6)	
Chips		38 (11.9)	13 (9.3)	25 (13.7)	
Sugamated drinks		10 (3.1)	4 (2.9)	6 (3.3)	
Table sugar intake	380				< 0.001
< 2 times a day		93 (24.5)	60 (33.9)	33 (16.3)	
≥ 2 times a day		105 (27.6)	34 (19.2)	71 (35.0)	
Do not take sugar		182 (47.9)	83 (46.9)	99 (48.8)	
**Oral hygiene practices**					
Child clean teeth	380	100 (26.3)	39 (22.0)	61 (30.0)	0.177
Onset of teeth cleaning	100				0.616
12–23 months		23 (23.0)	10 (25.6)	13 (21.3)	
24–36 months		77 (77.0)	29 (74.4)	48 (78.7)	
Child teeth cleaning methods	100				0.155
Fluoride toothpaste		55 (55.0)	18 (46.2)	37 (60.7)	
Clean cloth		45 (45.0)	21 (53.8)	24 (39.3)	
Child teeth cleaning frequency	100				0.363
Once a day		86 (86.0)	32 (82.1)	54 (88.5)	
Twice or more a day		14 (14.0)	7 (17.9)	7 (11.5)	
Child need support for cleaning	100	84 (84.0)	34 (19.2)	50 (24.6)	0.166
Mothers have dental visit experience	380	267 (70.3)	128 (72.3)	139 (68.5)	0.414
Mothers’ own teeth cleaning	380				< 0.001
Once a day		264 (69.5)	102 (57.6)	162 (79.8)	
≥ 2 times a day		75 (19.7)	49 (27.7)	26 (12.8)	
Never clean		41 (10.8)	26 (14.7)	15 (7.4)	

*p*-values are based on the χ^2^ test of association

### The prevalence of dental caries

**Fig. 1:**
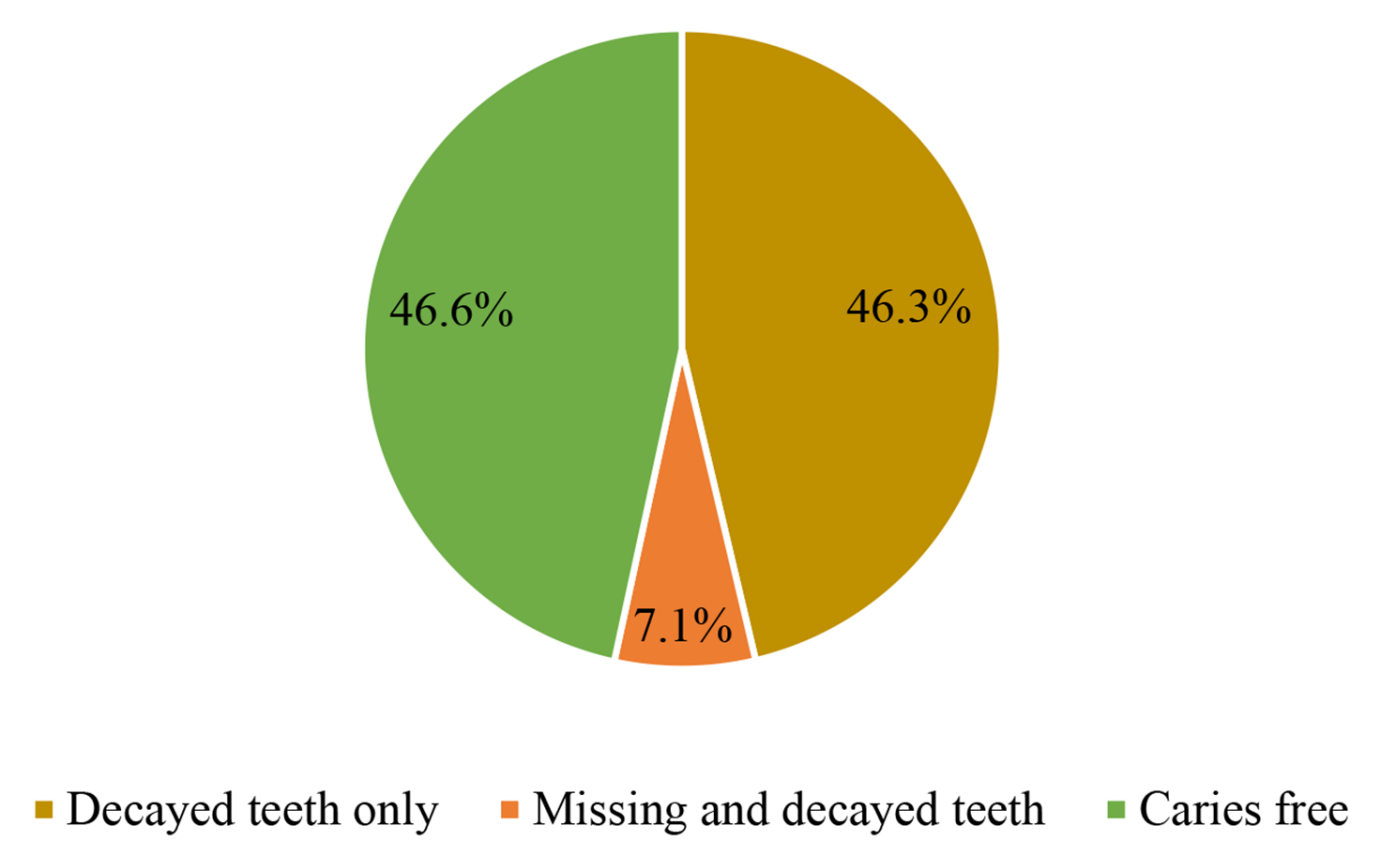
Magnitude of deft index among children 12–36 months old in Addis Ababa, Ethiopia, 2023

The prevalence of dental caries was 53.4% (95% CI: 48.3–58.5%) – 13.7% high caries (deft score ≥ 4) and 39.7% low caries (deft score 1–4). The decayed component constituted the largest share (46.3%) for the deft caries index. None of the children had their teeth filled at the time of the study (Fig. 1).

### Predictors of dental caries

Breastfeeding duration was not significantly associated with dental carries, after adjusting for confounders. However, the confounders – sugar consumption, child age, mothers’ employment, and marital status were significant predictors of dental carries.

Accordingly, children who consume sugar ≥ 2 times a day had 2.34 times higher odds to develop caries (OR = 2.34; 95% CI: 1.27, 4.31) compared to children who do not consume sugar. Older children, 24–36 months old, are 2.44 times more likely to develop dental caries than younger children, 12–23 months old (OR = 2.44; 95% CI: 1.42, 4.22). Children of unemployed mothers had 1.95 times higher odds of developing high caries compared to children of employed mothers (OR = 1.95; 95% CI: 1.05, 3.61). Children of not married women had 2.77 times higher odds of developing dental caries (OR = 2.77; 95% CI: 1.58, 4.86) compared to children of married women (Table 4).

**Table 4:** Crude and adjusted ORs and their 95% CIs of the predictors of dental caries among children 12–36 months old in Addis Ababa, Ethiopia, 2023

Characteristics	Crude OR, 95% CI			Adjusted OR, 95% CI		
	OR	95% CI	*p*-value	OR	95% CI	*p*-value
Breastfeeding duration						
< 6 months	Ref.			Ref.		
6–11 months	0.59	(0.30, 1.82)	0.138	0.48	(0.22, 1.08)	0.076
12–24 months	1.26	(0.77, 2.07)	0.350	1.08	(0.61, 1.90)	0.800
> 24 months	0.70	(0.39, 1.23)	0.214	0.63	(0.32, 1.24)	0.181
Feeding type						
Breastfeeding	1.76	(1.06, 2.91)	0.028	1.97	(1.06, 3.65)	0.310
Bottle-feeding	Ref.			Ref.		
Sweet snacking						
1–2 times a day	1.65	(0.90, 3.03)	0.105	1.45	(0.71, 2.97)	0.304
≥ 3 times a day	2.74	(1.49, 5.03)	0.001	1.87	(0.88, 3.98)	0.101
Do not snack	Ref.			Ref		
Table sugar consumption						
< 2 times a day	0.46	(0.27, 0.77)	0.003	0.90	(0.48, 1.68)	0.742
≥ 2 times a day	1.75	(1.06, 2.89)	0.029	2.34	(1.27, 4.31)	0.006
Do not take sugar	Ref.			Ref		
Mothers’ own teeth cleaning						
Once a day	2.75	(1.39, 5.44)	0.004	2.16	(0.99, 4.73)	0.053
≥ 2 times a day	0.92	(0.42, 2.03)	0.836	0.72	(0.29, 1.82)	0.493
Never clean	Ref.			Ref.		
Age of children						
12–23 months	Ref.			Ref.		
24–36 months	2.36	(1.56, 3.56)	< 0.001	2.44	(1.42, 4.22)	0.001
Mothers’ education						
No formal education	0.93	(0.50, 1.71)	0.805	0.80	(0.35, 1.85)	0.603
Primary or secondary	1.73	(1.10, 2.72)	0.017	1.58	(0.84, 3.00)	0.157
Technical level and above	Ref.			Ref.		
Mothers’ employment						
Unemployed	2.31	(1.44, 3.70)	< 0.001	1.95	(1.05, 3.61)	0.034
Employed (formal or informal)	Ref.			Ref.		
Current marital status						
Married	Ref.			Ref.		
Not married	2.83	(1.76, 4.55)	< 0.001	2.77	(1.58, 4.86)	< 0.001
Household monthly income (Birr)						
2,000–4,999	2.04	(1.17, 3.55)	0.012	0.87	(0.38, 1.98)	0.732
5,000–9,999	2.1	(1.26, 3.51)	0.005	1.09	(0.56, 2.13)	0.800
10,000 or more	Ref.			Ref.		

Ref.: Reference; OR: Odds Ratio; 95% CI: 95% confidence interval

## Discussion

Dental caries in young children is prevalent and one of the most common childhood diseases. It can start early in life and have both immediate and long-term consequences. It can interfere with comfort, nutrition, and school participation, which can negatively impact growth and development. Breastfeeding provides several benefits for children and infants, including optimal nutrition which decreases the risk of infections (gastrointestinal, respiratory, and ear), diarrheal diseases, and infant deaths [6, 18, 21]. However, evidence linking dental caries and its predisposing factors, particularly breastfeeding duration, among young children are scarce in Ethiopia. We conducted a facility-based study to estimate the prevalence of dental caries and its association with breastfeeding duration among children aged 12–36 months in Addis Ababa.

We found that the prevalence of dental caries in primary teeth among children aged 12–36 months in Addis Ababa was 53.4% (95% CI: 48.3, 58.5%). This figure is higher than the prevalence reported in other studies. For example, an Indian study reported a prevalence of 21.0% among children aged 12–36 months [33], a Tanzanian study reported a prevalence of 3.7% among children aged 6–36 months [5], and a Ugandan study reported a prevalence 17.6% among children aged 6–36 months [5]. The Global Oral Health Status Report indicates that the prevalence of dental caries among children 1–9 years old in Ethiopia was 42.3% [25]. The possible explanations for the high prevalence of dental caries in our study could be the high consumption of sweets, poor oral cleaning practices and dental care utilization among young children. Dental diseases are most preventable and can be treated at an early age or stage. However, lack of oral health policies and strategies, inadequate integration of oral health into primary health care, low coverage of oral health services, shortage of oral health workforce and infrastructure, and low awareness and demand for oral health care among the public are the major challenges for oral health care in Ethiopia [25].

Although breastfeeding duration contributed to dental caries development in the bivariate analysis, this association disappeared when we control for confounding factor in the Binary logistic regression. This finding aligns with studies conducted in Thailand, where prolonged breastfeeding was not associated with dental caries [21]. However, other studies have reported an association between dental caries and prolonged breastfeeding [15, 17, 18, 20]. To establish concrete evidence in the association between duration of breastfeeding and dental caries, further studies using longitudinal data or meta-analysis are warranted. The confounders, sugar consumption, child age, mothers employment, and marital status were significantly associated to the development of dental caries in our study. These factors are also reported in similar studies [5–7, 33].

Consumption of sugar increased the odds of caries development. This is consistent with studies conducted in Dessie Town, Ethiopia [24], in Uganda and Tanzania [5], in USA among American Indian children [6], and in Saudi Arabia [7]. Moreover, a recent evidence synthesis confirmed that both the amount and frequency of sugar consumption is a risk factor for dental caries development [2]. Older children, 24–36 months old, had higher odds of developing caries than the younger ones. A study conducted in India reported similar findings where older age of the child was a risk factor for caries development [33]. This can be explained by the fact that older children are more likely to consume sugar and sweets, which is a risk factor for caries development in our study as well as others [2, 5–7]. Children of not married women and unemployed mothers had higher odds of developing caries. These mothers are more likely to lack information about the risk factors of dental caries, including sugar and sweets consumption [5]. Evidence indicates that poor and disadvantaged population groups are indeed at higher risk of dental caries [1]. Promotion of healthy diet, especially limiting intake of sugar and sweets, and integrating oral health into the primary health platform are recommended.

Our findings should be interpreted with caution, as this study has some limitations. The cross-sectional design of the study could not allow us to establish causality between dental caries and breastfeeding duration. Our study is a facility-based and thus may not represent the general population. Some potential confounding factors, such as maternal oral bacterial level and genetic influence, were not accounted for. Although we used a standard tool for data collection, recall bias is unavoidable for those indicators measured retrospectively.

## Conclusions

We found that the prevalence of dental caries among children 12–36 months old in Addis Ababa was 53.4%. Duration of breastfeeding was not associated with dental caries, after adjusting for confounders. The confounders – sugar consumption, child age, mothers employment, and marital status – were significantly associated with the development of dental caries. Promotion of healthy diet, especially limiting intake of sugar and sweets, and integration of oral health into primary health care programs are recommended. Lastly, further research using longitudinal design or meta-analysis is recommended to establish more concise evidence on the association between breastfeeding duration and dental caries.

## Data Availability

All data generated or analyzed during this study are included in this published article.

## Abbreviations

CDC: Center for Disease Control and Prevention
CI: Confidence Interval
DHS: Demographic Health Survey
DEFT: Decayed, Extracted, and Filled Teeth
SD: Standard Deviation
OR: Odd Ratio
UNICF: United Nations International Children Fund
VIF: Variance Inflation Factor
WHO: World Health Organizations
